# Etiology, clinical characteristics and coinfection status of bronchiolitis in Suzhou

**DOI:** 10.1186/s12879-021-05772-x

**Published:** 2021-02-01

**Authors:** Jiahong Tan, Jinfeng Wu, Wujun Jiang, Li Huang, Wei Ji, Yongdong Yan, Meijuan Wang, Xuejun Shao

**Affiliations:** 1 Department of Respiratory Medicine, Children’s Hospital of Soochow University, Suzhou, China; 2 Children’s Hospital of Wujiang District, Suzhou, China; 3 Department of Clinical Laboratory, Children’s Hospital of Soochow University, Suzhou, China

**Keywords:** Etiology, Bronchiolitis, Infant, Respiratory syncytial virus, Coinfection

## Abstract

**Background:**

Bronchiolitis is a clinical syndrome commonly encountered in practice, particularly among infants and young children. To investigate the prevalence of pathogens in hospitalized children with bronchiolitis and study the clinical characteristics of bronchiolitis with or without coinfections.

**Methods:**

We investigated the respiratory specimens and clinical data of 1012 children with bronchiolitis who were treated at the Children’s Hospital of Soochow University between November 2011 and December 2018. The nasopharyngeal aspirates were examined to detect viruses by direct immunofluorescence assay or polymerase chain reaction (PCR). *Mycoplasma pneumoniae* (MP) was tested by PCR and enzyme-linked immunosorbent assay.

**Results:**

Of the 1134 children less than 2 years with bronchiolitis, 122 were excluded by exclusion criteria. Causative pathogen was detected in 83.2% (842 of 1012). The majority of these (614 [72.9%] of 842) were single virus infection. The most common pathogens detected were respiratory syncytial virus (RSV) (44.4%), MP (15.6%), and human rhinovirus (HRV) (14.4%). Coinfection was identified in 13.5% (137 of 1012) of the patients. Coinfection included mixed virus infection and virus infection with MP infection. Children with single virus infection had a higher rate of oxygen therapy compared with single MP infection.

**Conclusions:**

The most common pathogen detected in children with bronchiolitis is RSV, followed by MP and HRV. Coinfection leads to a longer period of illness, increased severity of the symptoms and increased risk of hypoxemia.

## Background

Bronchiolitis is an acute infection of the lower respiratory tract, particularly affecting the terminal and respiratory bronchioles, with the possibility of extending to the adjacent alveolar ducts and spaces [1, 2]. Bronchiolitis is the most frequent disease in children less than 2 years and is the leading cause of hospital admissions in this age group. Bronchiolitis is a well-known clinical entity, which affects around 1–3% of all healthy children [3], and the mortality rate reported for this condition is reported to be approximately 2 per 100,000 infants [4]. However, the mortality rate of bronchiolitis has even been reported to be 3.5% in China [5], which we should pay more attention on.

Respiratory syncytial virus (RSV) is the most common viral pathogen identified in children with globally, acute lower respiratory infection (ALRI); about 45% of hospital admissions and in-hospital deaths due to RSV-ALRI occur in children younger than 6 months [6, 7].Also, RSV is the most common pathogen identified in bronchiolitis, followed by parainfluenza virus (PIV) and adenovirus (ADV) [8, 9]. Furthermore, recent studies have determined that bacterial pathogens, particularly MP and *Chlamydophila pneumoniae* (CP), are responsible for bronchiolitis in children under 2 years of age [10–12]. However, the clinical relevance of the various pathogens involved in children still remains unclear.

In this study, we sought to evaluate the distribution of pathogens responsible for bronchiolitis in children ≤2 years of age and analyze the differences in the clinical features of bronchiolitis caused by different pathogenic agents, and explore the difference between simple infection and coinfection.

## Methods

### Subjects

We conducted a retrospective analysis of the data of 1012 children who were admitted to the Children’s Hospital of Soochow University for the management of bronchiolitis between November 2011 and December 2018. Children’s Hospital of Soochow University is a tertiary referral center at Jiangsu Province, East China. It has over 1000 beds and 50,000 inpatients annually. The inclusion criteria for this study were children aged between 1 month and 2 years, occurrence of first episode of wheezing, and clinical evidence of bronchiolitis (tachypnoea, wheeze, prolonged expiratory phase, and crackles on auscultation). The exclusion criteria: children had immunodeficiency, history of a diagnosis of chronic lung disease, or congenital heart disease. This study protocol was approved by the Medical Ethics Committee of Soochow University.

### Specimen collection

Within 24 h of admission, nasopharyngeal aspiration was performed to collect specimens from all patients. For aspiration, a suction catheter was used introduced through the nose and advanced into the lower portion of the pharynx, up to a distance of 7–9 cm. Nasopharyngeal aspirate was collected and sent for histopathological analysis within 30 min of collection. The retrieved sample was centrifuged (500×g, 10 min) and suspended in 2 mL saline and separated into 2 aliquots for direct immunofluorescence assay (DFA) and polymerase chain reaction (PCR) to identify pathogens.

### Microbe detection

A quantitative diagnostic kit (provided by Sun Yat-sen University Daan Gene Co., Ltd.) for MP DNA was performed to identify the 16 s rRNA gene of MP extracted from nasopharyngeal specimens [13]. DFA was performed to detect RSV, influenza virus (IV), PIV, and ADV. The assay kits were obtained from Chemicon (USA), and all staining procedures were performed according to the manufacturer’s instructions. Immunofluorescence studies were then conducted (Leica 020–518.500, Germany). RNA was extracted from the specimens using Trizol reagent (Invitrogen, USA), followed by cDNA synthesis by reverse transcription. The cyclic temperature settings were 94 °C for 30s; 55 °C for 30s; followed by 68 °C for 30s; and, after 45 amplification cycles, a final extension was performed at 68 °C for 7 min. For detection of human metapneumovirus (hMPV) and HRV fluorescent real-time PCR (BIO-RAD iCycler) was performed. DNA extraction and real-time PCR were to detect HBoV.

### Data collection

The medical records of the patients were reviewed and data regarding the following parameters were recorded: (1) demographic and clinical characteristics, including age, gender, and duration of symptoms prior to admission; (2) results of viral diagnostic tests performed in nasopharyngeal aspirates; (3) results of blood tests for inflammatory indices, including white blood cell (WBC) count, percentage of neutrophils, serum C-reactive protein (CRP) levels. Tachypnea was defined as follows: > 60 breaths/min in children < 2 months, > 50 breaths/min in children 2–11 months, > 40 breaths/min in children 12- < 24 months.

### Statistical analysis

All statistical analyses in this study were performed using the Statistical Package for the Social Sciences (version 25.0). Frequency distributions and rates were used for descriptive analyses. Parameters regarding the patient’s demographic data and baseline characteristics were analyzed using means (SD) or medians (25th–75th percentiles). Normal distribution was met by the data, as confirmed by using t-test variance analysis. For parameters with the non-normal distribution of data, the Kruskal-Wallis H test was used. Posthoc multiple comparisons were performed to determine the origins of significant differences, and the results were adjusted by using the Bonferroni method. *P* value of < 0.05 was considered to indicate statistical significance.

## Results

### Patients

**Fig. 1:**
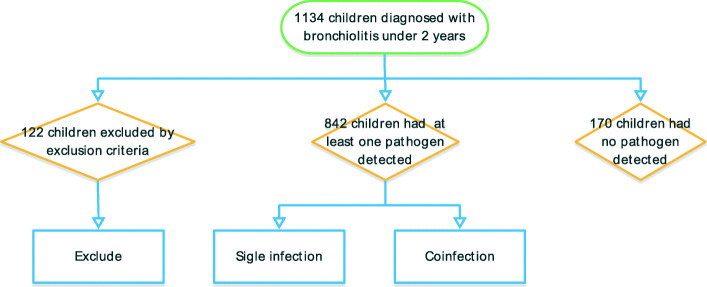
Study flow chart

Among the 1012 children with bronchiolitis identified in this study, one pathogen was detected in 842 (83.2%) children (Fig. 1). Among these patients, 603 (71.6%) were male and 239 (28.4%) were female, with ages ranging from 1 month to 24 months (median: 5 months). The median duration of symptoms before admission was 6 days. With respect to clinical presentation, 372 (44.2%) had sneezing, 241 (28.6%) had fever, 172 (20.4%) had tachypnea, 51 (6.1%) had dyspnea, and 164 (19.5%) required oxygen administration.

### Etiology

**Fig. 2:**
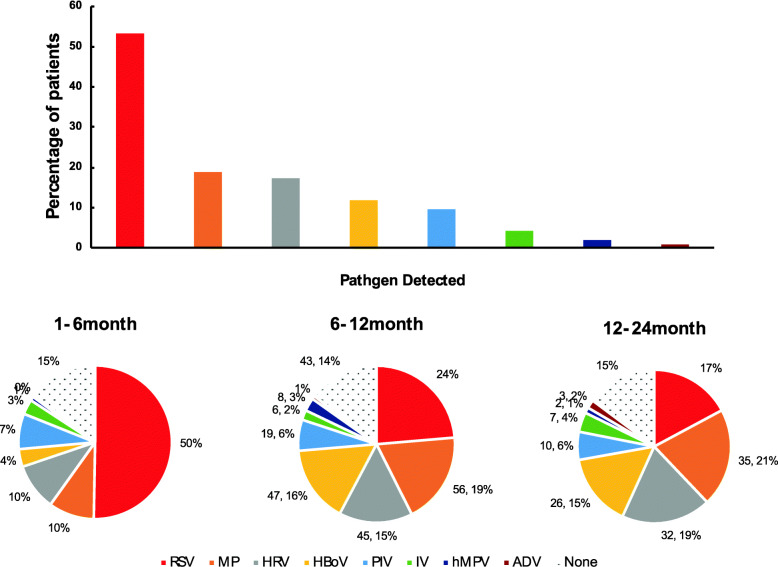
Distribution of pathogens at different age groups

The most common pathogens detected were RSV (44.4%), MP (15.6%), HRV (14.4%), HBoV (9.8%), and PIV (8.0%). Coinfection was identified in 137 (13.5%) of the patients. The viruses detected in coinfections were RSV, IV, HRV, HBoV, PIV, hMPV, and ADV. Further, among the patients, 62.3% were ≤ 6 months old; 24% were 6 months to ≤1 years old and 13.7% of patients were 1 to 2 years old. The most common pathogens isolated were RSV (58.9%), HRV (11.6%), MP (11.3%), and PIV (8.8%) in patients aged ≤6 months. On other hand, in the patients of ages between 6 months and ≤ 1 years, the most common pathogens were RSV (27.7%), MP (22.1%), HBoV (18.6%), and HRV (17.8%). For 1 to 2-year-old children, the most common pathogens were MP (24.3%), HRV (22.2%), RSV (20.1%), and HBoV (18.1%). Thus, for children under 6 months of age, RSV was identified as the most common pathogen responsible for bronchiolitis, while MP infection was less common (all *P* < 0.002) (Fig. 2).

### Mixed infections

**Fig. 3:**
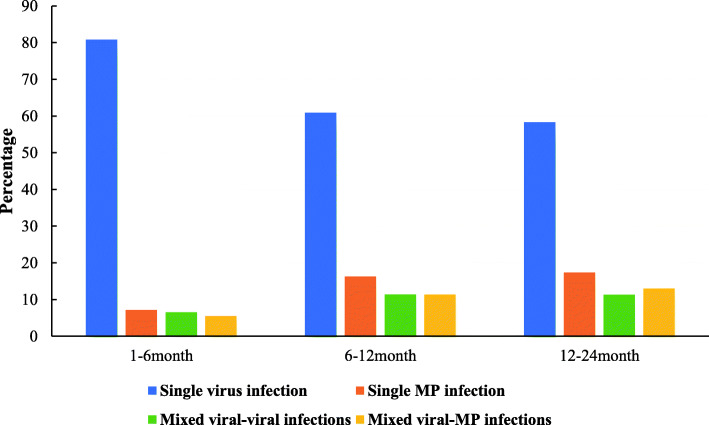
The proportion of infants with the single infection and coinfections

Of the 842 patients, 614 (72.9%) had single viral infections; 91 (10.8%) had only MP infections; 70 (8.3%) had multiple viral infections, and 67 (8.0%) had viral infection mixed with MP. Among patients aged ≤6 months, 12% had coinfection, with 6.5% having multiple viral infections and 5.5% having coinfection of a virus and MP. Among patients between 6 months and ≤ 1 years of age, 22.8% had coinfection, with 11.4% having mixed viral-viral infections and 11.4% having viral-MP infections. In the patients aged 1 to 2 years, 24.3% had coinfection, with 11.3 and 13.0% having mixed viral-viral and viral-MP coinfections, respectively. The probability of coinfection in children of age ≤ 6 months was significantly lower than in children of age between 6 months and ≤ 2 years (all *P* < 0.002) (Table 1 and Fig. 3).

**Table 1:** Pathogens Identified in Hospitalized Children with Bronchiolitis

Pathogen	No. of Episodes			Total No. of Episodes
	Single Infection	Coinfection With Viruses	Coinfection With MP	
Viruses ^a^				
RSV	377 (84.0)	43 (9.6)	29 (6.4)	449
HRV	86 (58.9)	43 (29.5)	17 (11.6)	146
HBoV	58 (58.6)	29 (29.3)	12 (12.1)	99
PIV	61 (75.3)	10 (12.3)	10 (12.3)	81
IV	16 (45.7)	15 (42.9)	4 (11.4)	35
hMPV	12 (75.0)	1 (6.3)	3 (18.8)	16
ADV ^b^	4	2	1	7
Atypical pathogen ^a^				
MP	91 (57.6)	67 (42.4)	–	158

^a^ Data are n (%)

^b^ The percentages are not listed because the total episodes is too small

### Comparisons of clinical characteristics of the patients with single and mix infections

Comparison of the demographic and clinical features of the children shows that children with single virus infection was the youngest (mean age: 5.58 months). Further, the number of children presenting with fever and percentage of neutrophils were the lowest among children infected with a single virus (all *P* < 0.001). The median duration of symptoms before admission of children with single MP infection (9 days), as well as those with viral-MP infection (7 days) was significantly greater than that of children infected with single virus (5 days) (*P* < 0.001, respectively). The mean length of stay of children with single virus infection (8.1 days), as well as those with viral-MP infection (8.0 days) was significantly less than that of children infected with viral-MP (9.0 days) (*P* < 0.05, respectively). Children with single virus infection had a higher rate of oxygen therapy compared with single MP infection (21.7% VS 8.8%, *P* = 0.004), while the PICU admission rate didn’t differ between the two groups (Table 2).

**Table 2:** The Demographic and Clinical Characteristics of 842 Patients with Bronchiolitis Associated with Single/Mixed infections

Characteristics	Single virus	Single MP	Mixed viruses	Mixed viruses/MP	*P* value
No. of patients	614	91	70	67	–
Age, months ^ac^	5.58	8.80	7.51	8.12	< 0.001
Gender, % male	72.5	67	74.3	67.2	0.560
Duration of symptoms before admission, days ^bd^	5	9	7	7	< 0.001
Length of stay ^b^	8	7	8	8	0.038
Fever, %^e^	23.6	40.7	35.7	50.7	< 0.001
Nasal congestion, %	45.8	31.9	41.4	49.3	0.082
Tachypnoea, %	21.5	13.2	20	17.9	0.303
Dyspnea, %	6.8	2.2	5.7	4.5	0.340
WBC count, × 10^9^/L ^a^	9.77	10.20	9.72	10.92	0.159
Percentage of neutrophils ^af^	33.3	39.05	38.46	40.78	< 0.001
CRP, mg/L^b^	0.39	0.76	0.34	0.96	0.133
Need of oxygen, %^g^	21.7	8.8	11.4	22.4	0.008
PICU admission, %	10.6	5.5	10	7.5	0.429

^a^ The mean value was used

^b^ The median value was used

^c^Posthoc multiple comparisons between groups found that significant differences were observed between single virus and single MP group (5.58 vs 8.80), single virus and mixed viruses group (5.58 vs 7.51), single virus and mixed viruses/MP group (5.58 vs 8.12)

^d^Posthoc multiple comparisons between groups found that significant differences were observed between single virus and single MP group (5 vs 9), single virus and mixed viruses/MP group (5 vs 7)

^e^Posthoc multiple comparisons between groups found that significant differences were observed between single virus and single MP group (23.6 vs 40.7), single virus and mixed viruses group (23.6 vs 35.7), single virus and mixed viruses/MP group (23.6 vs 50.7)

^f^Posthoc multiple comparisons between groups found that significant differences were observed between single virus and single MP group (33.30 vs 39.05), single virus and mixed viruses group (33.30 vs 38.46), single virus and mixed viruses/MP group (33.30 vs 40.78)

^g^Posthoc multiple comparisons between groups found that significant differences were observed between single virus and single MP group (21.7 vs 8.8), single virus and mixed viruses group (21.7vs 11.4), single MP and mixed viruses/MP group (8.8 vs 22.4)

## Discussion

Bronchiolitis is the most frequent disease affecting children of age less than 2 years, and it is a leading cause of hospital admissions in this age group. In this study, we conducted a retrospective investigation of 842 children hospitalized with bronchiolitis in order to identify the distribution of pathogens and coinfection. We found that 83.7% of the children had a single pathogen infection, whereas 16.3% had coinfections. A cohort analysis showed that pneumonia, bronchitis, asthma, bronchiolitis, and URTI were significantly more common in males [14]; and males are approximately twice as likely to become hospitalized than females due to RSV infection [15]. A new retrospective study in Italy also shows that there is a higher incidence of bronchiolitis in boys than in girls [16]. Specific pathogenic mechanisms between RSV and gender need further exploration.

The most common pathogens identify in our study were RSV (53.5%), followed by MP (18.8%) and HRV (17.3%). A longitudinal prospective investigation conducted in the USA revealed that RSV was the causative pathogen for bronchiolitis in 76% of infants; this percentage is much higher than that observed in our study; however HRV was isolated in 18% of the their cases, which is similar to the rate of HRV infection noted in our study [17]. In an 11-year study in Spain, 62.7% of the patients (children ≤2 years of age with acute bronchiolitis) had RSV infections, which is a percentage slightly higher than that noted in our study [18]. In a similar retrospective Slovenian study of children under 2 years with bronchiolitis, RSV (57.5%), HRV (25.6%), and HBoV (18.4%) were identified as the most common pathogenic viruses [19]; their results were similar to ours in the case of RSV but higher in the case of HRV.

The current study indicated that a single viral infection (72.9%) was most common type of infection in children under 2 years of age with bronchiolitis. We noted that RSV was the most common virus isolated, especially in infants under 6 months of age. We also noted that the percentage of RSV infection gradually decreased with age, which suggests that younger infants are more vulnerable to RSV disease; this is consistent with the findings of previous studies [20, 21]. A retrospective cohort study indicated that the reduced exposure of pregnant women to RSV epidemic contributed to more severe RSV-induced bronchiolitis in children under 6 months of age [22]. Therefore, RSV-induced bronchiolitis is common in 6 months age; this may be associated with the circulation of antibodies that are not associated with RSV infection during pregnancy. Further investigations are necessary to identify specific susceptibility factors.

Further, in our study, MP was detected in 15.6% of our patients. A 2-year prospective study showed that 9.0% of the bronchiolitis in infants had a positive pathogen of MP [12]. Some researchers from Thailand have reported the MP infected rate of 8.2 [23]. These indicates that the importance of MP in bronchiolitis are increasing. In our study, oxygen therapy are less required of bronchiolitis with MP, and comparing MP with other viruses bronchiolitis, children with MP bronchiolitis don’t cause more severe symptoms. Jonathan M [24] reported that 1% of MP was detected in a multicenter study of children with severe bronchiolitis. A study in Turkey also reported that Children infected with CP and MP had less severe bronchiolitis than those infected with RSV [12]. Therefore, the study results are consistent with the symptoms described.

One hundred and thirty seven (16.3%) of our patients had infection due to two pathogens. Interestingly, the distribution of multiple viral coinfection and viral-bacterial infection was similar. The probability of coinfection in children ≤6 months old was significantly lower than that in children aged between 6 months and ≤ 2 years of age. A prospective study from Turkey identified that the rate of coinfection among children with acute bronchiolitis was 34.2%, which is higher than the percentage observed in our study [25]. Similarly, a study from Israel showed that the rate of coinfectin in infants with acute bronchiolitis was nearly 40%, which is also higher than that in our study [26]. A UK study reported an even higher percentage of 46% [27]. The discrepancies in the rate of coinfection in bronchiolitis may be attributed to differences in the pathogen detection methods and the type of pathogens isolated.

The impact of coinfection on the severity of bronchiolitis still remains questionable. A comprehensive review in London revealed that multiple viral infection was associated with the admission of infants to the pediatric intensive care unit for the management of severe bronchiolitis [28]. In contrast, some studies have shown that there is no correlation between the presence of multiple coinfections and severity of bronchiolitis [29]. A Taiwanese study also reported that different viral pathogens do not give rise to different clinical characteristics among children with bronchiolitis [30]. However, a Brazilian study revealed that both coinfection and RSV load influenced the prognosis of acute bronchiolitis in infants [31]. Our findings in this study indicated that the duration of symptoms and duration of hospitalization in cases of single virus infection were significantly less than those observed in case of combined viral and MP infection. Thus, we believe that coinfection can aggravate the disease. Further studies are necessary to confirm these associations.

Some of the most common reasons for admission due to bronchiolitis are hypoxia, requirement for supplemental oxygen, poor feeding, and respiratory disease [32–34]. Small airway obstruction and the resultant mucus plugs and edema are believed to play a crucial role in the pathogenesis of bronchiolitis. In our study, the requirement of oxygen was most frequent among patients having coinfection with both viral pathogens and MP and least frequent among those with single MP infection. Studies have shown that the high incidence of hypoxia associated with RSV infection may be predictive of a poorer outcome [35], which is consistent with our findings.

## Limitations

In our study, we included only patients with bronchiolitis who had undergone investigations for the detection of the pathogenic agents; among the patients, we did not enroll any patients with only bacterial infection. Immunofluorescence assays have variable and lower sensitivity (69.4%) compared with PCR. Further investigations that cover a wider range of clinical presentations are warranted.

## Conclusion

RSV was identified as the most common pathogen causing bronchiolitis in infants and young children, followed by MP and HRV. Coinfection with multiple pathogens leads to persistence of the disease for a longer period and increased severity of the symptoms. In particular, coinfection increases the risk of hypoxemia. Measures to increase awareness among healthcare personnel regarding the disease and its complications are necessary.

## Data Availability

The datasets used and/or analyzed during the current study available from the corresponding author on reasonable request.

## Abbreviations

PCR: Polymerase chain reaction
MP: Mycoplasma pneumoniae
RSV: Respiratory syncytial virus
CP: Chlamydophila pneumoniae
DFA: Direct immunofluorescence assay
IV: Influenza virus
PIV: Parainfluenza virus
ADV: Adenovirus
hMPV: Human metapneumovirus
HRV: Human rhinovirus
WBC: White blood cell
CRP: C-reactive protein
SD: Standard deviation
